# Case Studies in Physiology: Male to female transgender swimmer in college athletics

**DOI:** 10.1152/japplphysiol.00751.2022

**Published:** 2023-03-17

**Authors:** Jonathon W. Senefeld, Sandra K. Hunter, Doriane Coleman, Michael J. Joyner

**Affiliations:** ^1^Department of Anesthesiology and Perioperative Medicine, Mayo Clinic, Rochester, Minnesota, United States; ^2^Department of Physiology and Biomedical Engineering, Mayo Clinic, Rochester, Minnesota, United States; ^3^Exercise Science Program, Department of Physical Therapy, Marquette University, Milwaukee, Wisconsin, United States; ^4^Athletic and Human Performance Research Center, Marquette University, Milwaukee, Wisconsin, United States; ^5^Duke University School of Law, Durham, North Carolina, United States

**Keywords:** cross-sex hormone therapy, gender-affirming hormone therapy, longitudinal analysis, sex differences, transsexual

## Abstract

There is current scientific and legal controversy about sports competition eligibility regulations for transgender athletes. In this case study, we quantified performances by an elite, transgender woman (male sex, female gender identity) college swimmer who competed in both the men’s and women’s National Collegiate Athletic Association (NCAA) categories. We also contextualized her performances with respect to world-record performances and contemporary elite college swimmers. These data demonstrate that the declines in freestyle swimming performances of a transgender woman after about 2 yr of reported feminizing gender-affirming hormone treatment (0.5% for the 100 to 7.3% for the 1,650 yard distance) are smaller than the observed sex-related differences in performance of top 200 world record performances in metric distances of similar durations (11.4% for the 100 m to 9.3% for the 1,500 m distance). Despite slower performances, the transgender woman swimmer experienced improvements in performance for each freestyle event (100 to 1,650 yards) relative to sex-specific NCAA rankings, including producing the best swimming time in the NCAA for the 500-yard distance (65^th^ in the men’s category in 2018–2019 to 1^st^ in the women’s, 2022). Similarly, NCAA-ranked male swimmers had no improvements in rank in the men’s category during the same time frame. Our findings suggest that the performance times of the transgender woman swimmer in the women’s NCAA category were outliers for each event distance and suggest that the transgender woman swimmer had superior performances relative to rank-matched swimmers. Our analysis may be useful as a framework for regulators considering participation guidelines, which promote fair competition for all athletes—irrespective of gender identity.

**NEW & NOTEWORTHY** This case study, longitudinal analysis of freestyle swimming performances before and after 2 yr of feminizing gender-affirming hormone therapy of an elite transgender woman (male sex, female gender identity), demonstrates superior performance relative to rank-matched female swimmers and a lower performance gap than previously observed between elite male and female swimmers.

## Graphical abstract

**Figure:**
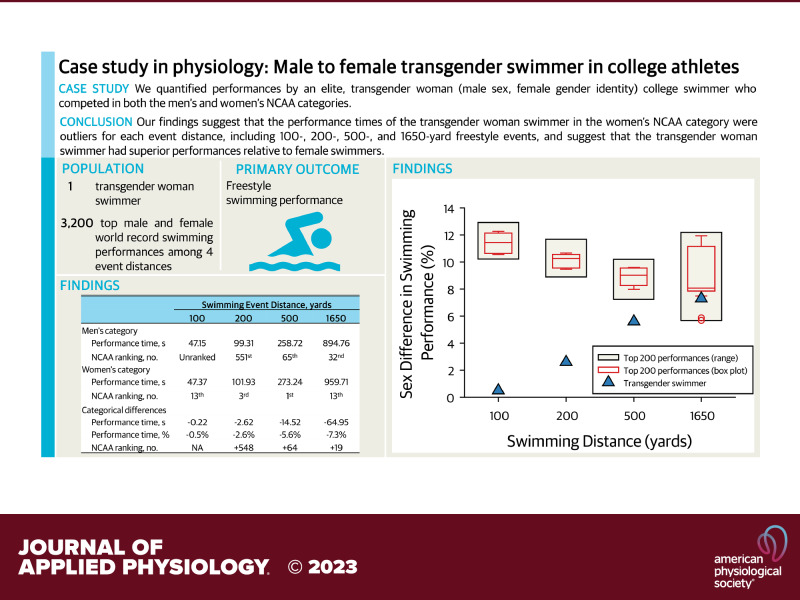

## INTRODUCTION

Recent outstanding performances by a transgender woman (male sex, female gender identity) swimmer have raised controversy about the role of gender-affirming hormone treatments on athletic performance and potential “legacy advantages” associated with male testosterone concentrations and male puberty (1, 2). Substantial evidence demonstrates that testosterone is strongly associated with sex-based differences in sports performance, more so than any other known factor (3–5). Male testosterone concentrations are, on average, 10 to 20 times greater than female testosterone concentrations (6). The pronounced differences between the sexes in endogenous testosterone levels through puberty correspond to sex-specific divergence in anatomical skeletal structure (e.g., limb length and height), skeletal muscle mass, strength and endurance, cardiac output, lung capacity, hematocrit, and, as a result of this divergence, athletic performance (3, 4, 6).

This sex-based dichotomy in testosterone is strong evidence supporting endogenous (or circulating) testosterone as a key biological marker to distinguish competitive cohorts in most sports, excluding those sports that are not determined by maximal skeletal muscle or cardiopulmonary performance (e.g., chess, electronic sports, or shooting sports) (7–9).

Thus, transgender women who experienced male puberty before starting feminizing gender-affirming hormone therapy (GAHT) likely have an athletic advantage over athletes who experienced female puberty (7, 10, 11). Although feminizing GAHT is associated with reductions in the factors that contribute to the male athletic advantage (including reductions in muscle strength and size, and hemoglobin concentration), the duration of feminizing GAHT required to mitigate the athletic advantage for transgender women may be 3 yr or more (12).

In this framework, endogenous testosterone is understood to be the primary determinant of the dichotomy in sport performance between male and female athletes. Thus, endogenous testosterone concentrations have been widely used since 2010 by international and domestic sports governing bodies, including the National Collegiate Athletic Association (NCAA), as both the proxy for male athletic advantage and the basis for eligibility for the female category. Specifically, the NCAA required that transgender women complete 1 yr or more of feminizing GAHT before competing on a women’s team. However, unlike other sports governing bodies, before January 2022, the NCAA did not establish a testosterone concentration above which an athlete-born male could not compete in the female category, nor did the NCAA actively monitor compliance with its nonspecific requirements.

In this context, there was controversy about whether 1 yr of feminizing GAHT was sufficient to mitigate the “legacy effects” associated with years of exposure to training with normal male endogenous testosterone concentrations. Thus, we quantified performances by an elite transgender college swimmer before and after publicly disclosed self-reported 24 consecutive months of feminizing GAHT. We contextualized these performance results with respect to theoretical predictions to determine the extent to which the recent performances by the transgender athlete might be statistical outliers. These analyses represented a proxy to estimate the “legacy effects” of endogenous testosterone and male puberty on the performance of elite transgender women receiving feminizing GAHT.

## MATERIALS AND METHODS

### Case Study

The primary subject of this case study was a transgender woman (male sex, female gender identity) who competed in men’s NCAA freestyle swimming from 2017 to 2020 and then women’s NCAA freestyle swimming in the 2021–2022 season. Notably, there was no 2020 NCAA Swimming Championships competition due to the COVID-19 pandemic. All procedures accessed public information and did not require ethical review as determined by the Mayo Clinic Institutional Review Board in accordance with the Code of Federal Regulations, 45 CFR 46.102, and the Declaration of Helsinki.

We compared swimming performance times of a transgender woman athlete while competing in the men’s category in NCAA Division I freestyle events (100, 200, 500, and 1,650 yards) before she began feminizing GAHT with those performance times while competing in the women’s category after 2 yr of GAHT. As detailed in the next three sections of the MATERIALS AND METHODS, subsequent comparisons were made to the top swimming performances of male and female swimmers, the trajectory of male and female swimmers over the 4-yr NCAA career, and male competitors of similar rank who competed against the swimmer in the male category.

### Comparator Data: Top Swimming Performances

To contextualize the case study, the top 200 male and female world record freestyle swimming performance times for both short course and long course for each of four event distances (100, 200, 400, and 1,500 m) were obtained. These event distances closely approximate NCAA imperial distances (100, 200, 500, and 1,650 yards). Because the focus of these analyses is on a case study of a swimmer in the NCAA, NCAA imperial distances are referenced throughout this article. Hence, 3,200 world records were abstracted (200 places × 2 courses × 2 sexes × 4 distances). Swimming times were downloaded from the international swimming federation’s (World Aquatics formerly known as FINA) database (https://www.fina.org/swimming/rankings) for world record times. We then paired the male versus female times (e.g., 1st vs. 1st, 2nd vs. 2nd, etc.) within each swimming distance and course, and calculated a percentage difference for each pair to derive a range of expected sex-based differences in swimming performance. Sex-based differences in swimming performance time were calculated for each event distance as [(male’s performance time) – (female’s performance time)] × (female’s performance time)^−1^ × 100%.

### Comparator Data: Trajectory to Top NCAA Performance

To contextualize the general progression to the top (number 1) NCAA Division I performance rank for any freestyle event (50, 100, 200, 500, or 1,650 yard distance), the best 1,000 times for each NCAA season were obtained including data from the 2010–2011 season to the 2021–2022 season. Swimming times from the short course yards pool were downloaded from the USA Swimming database (https://www.usaswimming.org/times/ncaa/ncaa-division-i). All male and female swimmers who were ranked 1st in NCAA Division I competition at the end of a season were included in these analyses, including 41 swimmers competing in the women’s category and 39 swimmers competing in the men’s category. Although theoretically these analyses could have included 120 swimmers (2 categories × 12 seasons × 5 events), the analyses only included 90 swimmers due to several swimmers obtaining number 1 ranking within a given event distance for multiple seasons.

### Comparator Data: Progression of Contemporary Male Swimmers

To contextualize the general progression of male swimmers who had a similar rank to the transgender woman swimmer in the 2018–2019 NCAA competition year (65th NCAA rank in the men’s category before gender transition), we examined the progression of male swimmers ranked 45th to 85th in the 500 yard distance during that NCAA competition year (2018–2019). These data were derived from the database previously described (top 1,000 times for NCAA seasons 2010–2011 to 2021–2022).

### Statistical Analyses

Data were reported as means ± SD within the text. Separate mixed-model univariate analyses of variance (ANOVAs) were used to compare the dependent variables (swimming velocity and relative performance (%1st place) of males and females, and sex differences in swimming velocity) between independent variables [competition category (men and women), event distance (100, 200, 500, and 1,650 yard) and eligibility year (1, 2, 3, and 4)]. Tukey’s method was used to identify far statistical outliers. Tukey’s method identifies values that are 3.0 IQR below Q1 and 3.0 IQR above Q3 as outliers, where IQR denotes inter quartile range, Q1 denotes lower quartile, and Q3 denotes upper quartile. In this context, outliers approximate the 99th or 1st percentile of a data set. Analyses were performed using IBM Statistical Package for Social Sciences version 28 statistical package (IBM, Armonk, NY). Statistical significance was set at α = 0.05, and all tests were two tailed. Figures were created using SigmaPlot software (SigmaPlot 12.5, Systat Software Inc., Chicago, IL).

## RESULTS

### Transgender Woman Swimmer Performance Changes

**Figure 1.**
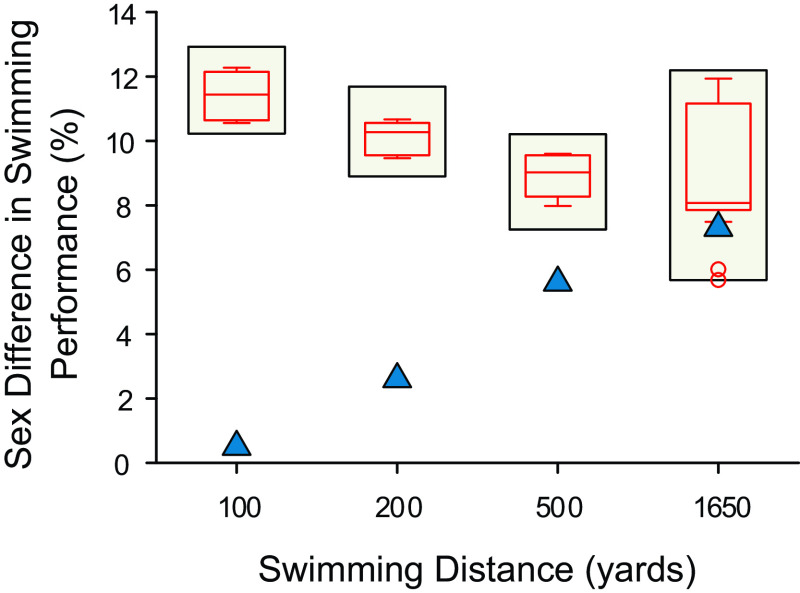
Differences in freestyle swimming performances between men’s and women’s categories for both a transgender woman (male sex, female gender identity) swimmer and top swimming performances of all time in the world. Span charts (beige bars) display the range of sex differences in swimming performance (%) observed in a dataset comparing male-vs.-female differences in top swimming performances of all time in the world. Each swimming distance represents 400 data points. Box plots (red lines) display the variance of the world’s best performances—horizontal lines in the middle of each box indicate the median; top, and bottom borders indicate the 75^th^ and 25^th^ percentiles; whiskers above and below the box indicate the 90^th^ and 10^th^ percentiles. Outliers beyond the ∼99^th^ or ∼1^st^ percentiles are represented as red circles. Scatter plots displaying the differences in performance time between men’s and women’s categories of a transgender woman swimmer are represented as blue triangles.

After about 2 yr of both feminizing GAHT and elite-level swimming training, swimming performance times by a transgender woman were slower by ∼5% across all relevant swimming event distances (Table 1 and Fig. 1). Although the small sample size of four observations may interfere with accurate inference from a correlation (13), there appears to be a linear relationship between performance times in the men’s category and the women’s category (*R*^2^ = 1.0, *P* < 0.001). The relationship between the performance times in the men’s category (before GAHT) and women’s category (after GAHT) can be described using the following equation: women’s category time = (men’s category time) × 0.9281 + 4.2546. The difference in performance times between the male category and female category also appeared to be associated with performance distance, such that the smallest decrement in performance was observed in the shortest event distance (0.5%, 100 yard) and the largest decrement in performance was observed in the longest event distance (7.3%, 1,650 yards; Table 1). Despite slower performances, the transgender woman swimmer experienced improvements in performance for each freestyle event (100 to 1,650 yards) relative to sex-specific NCAA rankings, including producing the best swimming time in the NCAA for the 500 yard distance (Table 1).

**Table 1.** Personal best freestyle swimming performances by a transgender woman (male-to-female transition) in NCAA college athletics in both men’s and women’s competition categories

	Swimming Event Distance, Yards			
	100	200	500	1,650
Men’s category				
Performance time, s	47.15	99.31	258.72	894.76
NCAA ranking, no.	Unranked^a^	551^st^	65^th^	32^nd^
Women’s category				
Performance time, s	47.37	101.93	273.24	959.71
NCAA ranking, no.	13^th^	3^rd^	1^st^	13^th^
Categorical differences^b^			
Performance time, s	−0.22	−2.62	−14.52	−64.95
Performance time, %	−0.5%	−2.6%	−5.6%	−7.3%
NCAA ranking, no.	NA	+548	+64	+19

^a^Unranked indicates that the performance was not within the fastest ∼600 performances of the season.

^b^The categorical differences represent the differences in performance of the transgender woman between men’s and women’s competition categories. Thus, data were calculated using 2018–2019 NCAA performance in the men’s category and 2021–2022 NCAA performance data in the women’s category. NA, not available; NCAA, NCAA, National Collegiate Athletic Association; no., number.

### Case Study Comparison With Top Swimming Performances of All Time

As a comparator group, we compared the performances of a transgender woman swimmer in both the men’s and women’s NCAA Division 1 categories to male-versus-female differences in top swimming performances of all time in the world. As expected, based on known physiological differences between the sexes and historical trends, males had faster performances than females in all swimming event distances (*P* < 0.001, Fig. 1) among the top male and female swimming performances of all time in the world. These sex-related differences between swimming performances of males and females were larger for shorter distance events (∼10.8%; 100 and 200 yards) and smaller for longer distance events (∼9.1%; 500 and 1,650 yards), as previously observed (14).

Comparing the recent case of the transgender woman swimmer with the historical comparator group of the world’s best athletes, we note that her recent performances in the women’s category (compared with her performance times in the men’s category before her gender transition) have smaller differences than comparator data. As a theoretical scenario, we added the within-swimmer changes in performance (comparison of her performance in the men’s category and women’s category) to the comparator group of top male and female athletes in the world. Using this theoretical approach, her within-swimmer changes were identified as statistical outliers for each event distance using Tukey’s method to identify outliers. Importantly, within the comparator data set, two additional statistical outliers were identified in the 1,650 yard event (5.69%, 5.87%). These data suggest that the decrements in performance of the transgender woman swimmer are less than expected based on a comparison of a large cohort of world class performances by female and male swimmers (Fig. 1), particularly for shorter event distances (100, 200, and 500 yards).

### Trajectory to Top NCAA Performance

**Figure 2.**
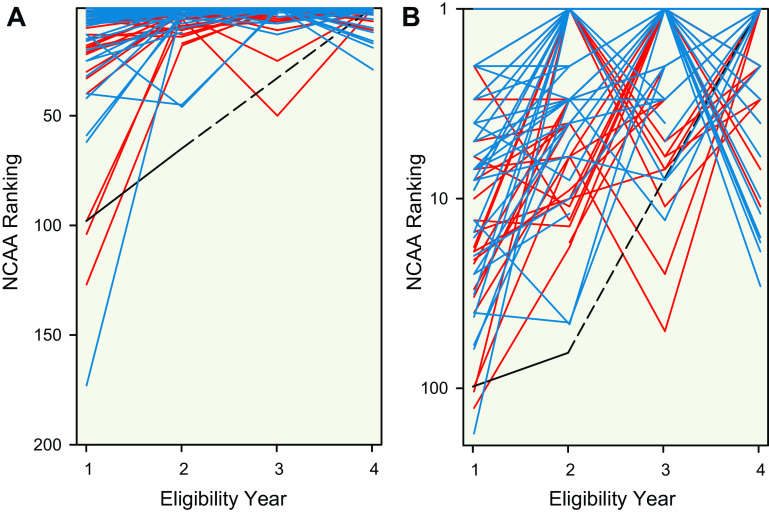
Trajectory to top NCAA swimming performance. Line plots display the trajectory across eligibility year of both male (blue lines) and female swimmers (red lines) who have been ranked first in an event distance (including all NCAA freestyle event distances) at the end of an NCAA season during a period of 12 seasons (2010–2011 to 2021–2022). The black lines represent trajectory of a transgender woman (male sex, female gender identity) who competed in both men’s and women’s NCAA freestyle swimming. Dotted lines are used to demonstrate that the transgender woman did not compete in her 3^rd^ year of eligibility due to following NCAA guidelines on gender-affirming hormone therapy. To provide multidimensional visual representation, panel *A* includes a linear scale for the *y* axis and panel *B* includes a semilog scale for the *y* axis. NCAA, National Collegiate Athletic Association.

As a theoretical comparator, we examined the trajectory across eligibility years of all swimmers (male and female) who have been ranked first in an event distance (including all NCAA freestyle event distances) at the end of an NCAA season for a period of 12 seasons (2010–2011 to 2021–2022; Fig. 2). Among this group of number one ranked swimmers, average annual NCAA rankings improved between first eligibility year (18.6 ± 30.7, mean ± SD) and second eligibility year (5.3 ± 8.1, *P* < 0.001). In the first eligibility year, six values were ranked below 58th place and were identified as statistical outliers. In the second eligibility year, six values were ranked below 14th place and were identified as outliers. Average ranking was not different between eligibility years two, three, and four (all post hoc tests, *P* ≥ 0.445). There were no sex-related differences in this change in ranking across NCAA eligibility years (main effect: *P* = 0.754, interaction: *P* = 0.884).

The transgender woman swimmer (case study) was ranked 1st in the 2021–2022 season for the 500-yard distance in the women’s category. For the 500 yard distance, the transgender woman swimmer progressed from the 98th and 65th place rankings in the men’s category during eligibility years one and two to the 1st place ranking in eligibility year four (Fig. 2). These performances of the transgender woman swimmer would be identified as outliers among swimmers who progressed to top NCAA performances.

### Progression of Contemporary Male Swimmers

**Figure 3.**
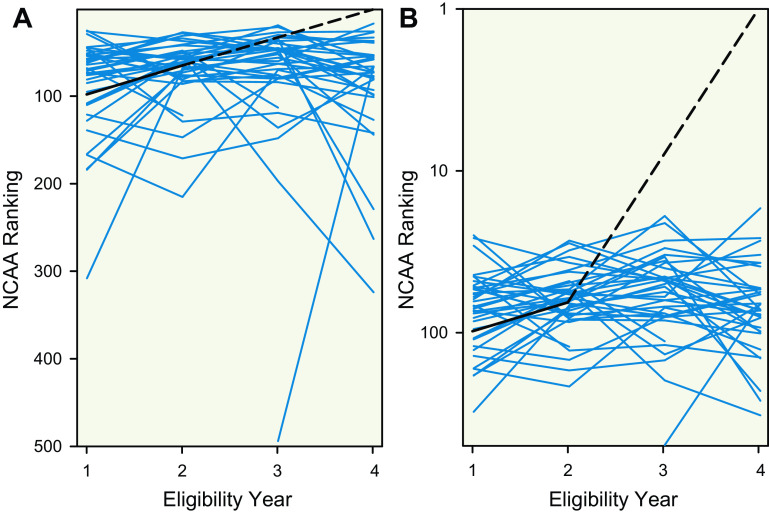
Progression of contemporary male swimmers and a transgender woman (male sex, female gender identity) swimmer for the 500-yard freestyle event. Blue line plots display the progression of contemporary male swimmers ranked 45^th^ to 85^th^ in the 500-yard freestyle distance in the 2018–2019 NCAA season, and the black line represents progression of a transgender woman swimmer who was ranked 65^th^ in the 500-yard freestyle distance in the 2018–2019 NCAA season. Dotted lines are used to demonstrate that the transgender woman did not compete in her 3^rd^ year of eligibility due to following NCAA guidelines on gender-affirming hormone therapy. To provide multidimensional visual representation, panel *A* includes a linear scale for the *y* axis and panel *B* includes a semilog scale for the *y* axis. NCAA, National Collegiate Athletic Association.

**Figure 4.**
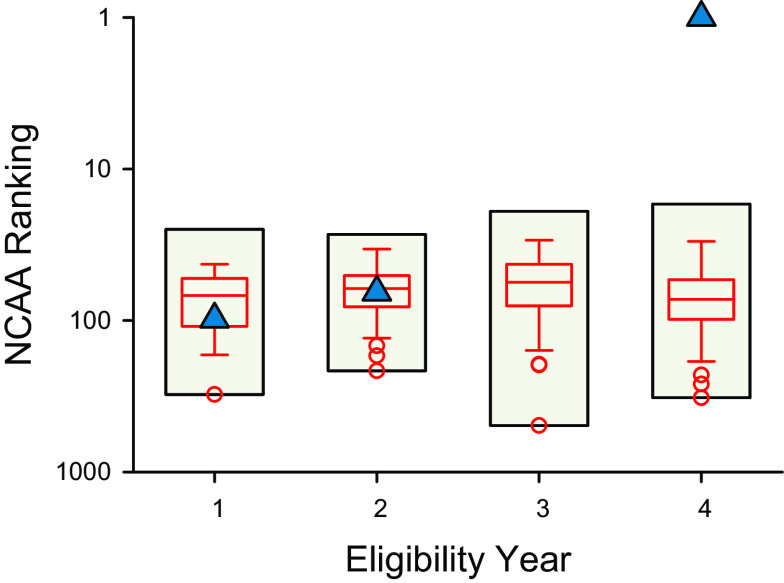
Progression of NCAA rankings for the 500-yard freestyle event among male swimmers and a transgender woman (male sex, female gender identity) swimmer. Span charts (beige bars) display the range of NCAA rankings observed in a dataset of swimmers ranked 45^th^ to 85^th^ in the 500-yard freestyle distance in the 2018–2019 NCAA season. Box plots (red lines) display the variance of the world’s best performances—horizontal lines in the middle of each box indicate the median; top, and bottom borders indicate the 75^th^ and 25^th^ percentiles; whiskers above and below the box indicate the 90^th^ and 10^th^ percentiles. Outliers beyond the ∼99^th^ or ∼1^st^ percentiles are represented as red circles. Scatter plots displaying the progression of NCAA rankings of a transgender woman swimmer are represented as blue. NCAA, National Collegiate Athletic Association.

As an additional theoretical comparator, we examined the progression of contemporary male swimmers in the 500 yard distance who had a similar rank (45th to 85^th^ rank) to the transgender woman swimmer (65th rank) in the 2018–2019 NCAA season (Figs. 3 and 4). Exploratory analyses of distributions of NCAA rank across eligibility years identified several outliers for each eligibility year, including year 1 (1 outlier, 308th rank), year 2 (3 outliers, ≥ 147th rank), year 3 (3 outliers, ≥ 195th rank), and year 4 (3 outliers, ≥ 229th rank). Notably, these outliers were all ‘high’ outliers demonstrating relatively “bad” performance rankings. However, there was only one “low” outlier observed (i.e., large improvement in ranking)—the transgender woman swimmer reaching NCAA rank 1 in eligibility year 4.

## DISCUSSION

In this case study, we examined the performance times of a transgender woman (male sex, female gender identity) who competed in both men’s and women’s NCAA freestyle swimming and contextualized her performances relative to the performances of both world class and contemporary NCAA swimmers. Our findings suggest that the performance times of the transgender woman swimmer were outliers in the women’s NCAA category and suggest that male puberty and training with exposure to male concentrations of testosterone likely conferred an athletic advantage despite about 2 yr of feminizing GAHT for the transgender woman swimmer. Our findings were supported by three separate analyses.

First, the declines in freestyle swimming performance for the transgender woman swimmer after about 2 yr of feminizing GAHT (0.5% for the 100 to 7.3% for the 1,650 yard distance) were less than sex differences observed among the top world record performances (11.4% for the 100 to 9.3% for the 1,650 yard distance) (Fig. 1). Second, despite slower performances, the transgender woman swimmer experienced improvements in performance for each freestyle event relative to sex-specific NCAA ranking, including improving from 65th rank to 1st rank for the 500 yard distance, and these improvements were identified as statistical outliers (Fig. 2). Third, the improvements of similarly ranked male swimmers (near 65th rank) were much less than the improvements observed for the transgender woman swimmer (Figs. 3 and 4). Notably, these findings revealed a consistent pattern among results arising from multiple different analyses whereby recent performances by a transgender woman swimmer were statistical outliers. These analyses suggest that among trained athletes there may be a prolonged legacy effect (greater than two years) associated with endogenous male testosterone concentrations or male puberty on freestyle swimming performances after feminizing GAHT.

Our findings suggest that the performance times of the transgender woman swimmer in the women’s NCAA category were outliers for each event distance, including 100-, 200-, 500-, and 1,650-yard freestyle events. These data suggest that the relative improvements in this swimmer’s rankings in the women’s category relative to the men’s category are likely due to “legacy effects” of endogenous testosterone and/or male puberty on a number of physiological factors that can influence athletic performance. Notably, the relative improvements in this swimmer’s rankings in the women’s category relative to the men’s category were greatest for shorter event distances (100, 200, and 500 yards), which are closely associated with anthropometrics, maximal skeletal muscle strength and power, and less dependent on hemoglobin concentrations.

In this context, our analysis may be useful as a framework for regulators considering participation guidelines for transgender athletes in the female category. It also adds to existing knowledge about the duration of the performance-enhancing effects of male concentrations of endogenous testosterone after feminizing GAHT has begun.

### Case Considerations

This case study focused on performance times from one transgender woman swimmer, which limits the mechanistic insights inferred from the primary results. Moreover, these analyses were limited by a lack of information on key performance-influencing factors before and after feminizing GAHT for the transgender woman swimmer, for example, training levels, circulating testosterone concentrations, and method and duration of testosterone blockade. Thus, these data should not be used to infer definitive effects of feminizing GAHT or “legacy effects” of testosterone and/or male puberty—these topics warrant further investigation.

### Conclusions

In summary, these data suggest there may be a prolonged “legacy effect” (greater than 2 yr) associated with endogenous male testosterone concentrations or male puberty on freestyle swimming performances after feminizing GAHT, particularly for shorter event distances (100, 200, and 500 yards), which are closely associated with anthropometrics and maximal skeletal muscle strength and power, and less dependent on hemoglobin concentrations. Accommodations should be made in sports to safeguard fairness for all athletes—whatever their gender identity—and will likely vary between sports and events at different levels of competition. While striving for fair and inclusive policies for all athletes, both the magnitude and duration of the profound influence of testosterone and male puberty on sports performance should be recognized with appropriate consideration.

## DATA AVAILABILITY

Data will be made available upon reasonable request.

## DISCLAIMERS

All publicly available information indicates that throughout her competitive career, the transgender woman swimmer adhered to applicable NCAA eligibility regulations. Sports-governing bodies, including the NCAA, are encouraged to ensure that their eligibility regulations continue to be based on the best available science and do not discriminate on the basis of gender identity. This case study is intended to provide additional evidence to contribute to objective policies for the fair integration of transgender athletes in sports.

## DISCLOSURES

No conflicts of interest, financial or otherwise, are declared by the authors.

## AUTHOR CONTRIBUTIONS

J.W.S., S.K.H., D.C., and M.J.J. conceived and designed research; J.W.S. analyzed data; J.W.S., S.K.H., D.C., and M.J.J. interpreted results of experiments; J.W.S. prepared figures; J.W.S., S.K.H., D.C., and M.J.J. drafted manuscript; J.W.S., S.K.H., D.C., and M.J.J. edited and revised manuscript; J.W.S., S.K.H., D.C., and M.J.J. approved final version of manuscript.

## References

[1] Devine C. Female Olympians’ voices: female sports categories and International Olympic Committee Transgender guidelines. Int Rev Soc Sport 57: 335–361, 2022. doi:10.1177/10126902211021559.

[2] Reynolds A, Hamidian Jahromi A. Transgender athletes in sports competitions: how policy measures can be more inclusive and fairer to all. Front Sports Act Living 3: 704178, 2021. doi:10.3389/fspor.2021.704178. 34337407 PMC8316721

[3] Handelsman DJ, Hirschberg AL, Bermon S. Circulating testosterone as the hormonal basis of sex differences in athletic performance. Endocr Rev 39: 803–829, 2018. doi:10.1210/er.2018-00020. 30010735 PMC6391653

[4] Senefeld JW, Clayburn AJ, Baker SE, Carter RE, Johnson PW, Joyner MJ. Sex differences in youth elite swimming. PLoS One 14: e0225724, 2019. doi:10.1371/journal.pone.0225724. 31756208 PMC6874329

[5] Howden EJ, Perhonen M, Peshock RM, Zhang R, Arbab-Zadeh A, Adams-Huet B, Levine BD. Females have a blunted cardiovascular response to one year of intensive supervised endurance training. J Appl Physiol (1985) 119: 37–46, 2015. doi:10.1152/japplphysiol.00092.2015. 25930024 PMC6345209

[6] Senefeld JW, Lambelet Coleman D, Johnson PW, Carter RE, Clayburn AJ, Joyner MJ. Divergence in timing and magnitude of testosterone levels between male and female youths. JAMA 324: 99–101, 2020. doi:10.1001/jama.2020.5655. 32633795 PMC7341166

[7] Roberts TA, Smalley J, Ahrendt D. Effect of gender affirming hormones on athletic performance in transwomen and transmen: implications for sporting organisations and legislators. Br J Sports Med 55: 577–583, 2021. doi:10.1136/bjsports-2020-102329. 33288617

[8] Wood RI, Stanton SJ. Testosterone and sport: current perspectives. Horm Behav 61: 147–155, 2012. doi:10.1016/j.yhbeh.2011.09.010. 21983229 PMC3264812

[9] Hamilton BR, Guppy FM, Barrett J, Seal L, Pitsiladis Y. Integrating transwomen athletes into elite competition: the case of elite archery and shooting. Eur J Sport Sci 21: 1500–1509, 2021. doi:10.1080/17461391.2021.1938692. 34077312

[10] Alvares LAM, Santos MR, Souza FR, Santos LM, Mendonça BB, Costa EMF, Alves M, Domenice S. Cardiopulmonary capacity and muscle strength in transgender women on long-term gender-affirming hormone therapy: a cross-sectional study. Br J Sports Med 56: 1292–1298, 2022 [Erratum in *Br J Sports Med* 57: e2, 2023]. doi:10.1136/bjsports-2021-105400. 36195433

[11] Hamilton BR, Lima G, Barrett J, Seal L, Kolliari-Turner A, Wang G, , et al. Integrating transwomen and female athletes with differences of sex development (DSD) into Elite Competition: The FIMS 2021 Consensus Statement. Sports Med 51: 1401–1415, 2021 [Erratum in *Sports Med* 51: 1417–1418, 2021]. doi:10.1007/s40279-021-01451-8. 33761127 PMC7988249

[12] Harper J, O'Donnell E, Sorouri Khorashad B, McDermott H, Witcomb GL. How does hormone transition in transgender women change body composition, muscle strength and haemoglobin? Systematic review with a focus on the implications for sport participation. Br J Sports Med 55: 865–872, 2021. doi:10.1136/bjsports-2020-103106. 33648944 PMC8311086

[13] Jenkins DG, Quintana-Ascencio PF. A solution to minimum sample size for regressions. PLoS One 15: e0229345, 2020. doi:10.1371/journal.pone.0229345. 32084211 PMC7034864

[14] Millard-Stafford M, Swanson AE, Wittbrodt MT. Nature versus nurture: have performance gaps between men and women reached an asymptote? Int J Sports Physiol Perform 13: 530–535, 2018. doi:10.1123/ijspp.2017-0866. 29466055

